# Backtracking and forward checking of human listeriosis clusters identified a multiclonal outbreak linked to *Listeria monocytogenes* in meat products of a single producer

**DOI:** 10.1080/22221751.2020.1784044

**Published:** 2020-07-12

**Authors:** Stefanie Lüth, Sven Halbedel, Bettina Rosner, Hendrik Wilking, Alexandra Holzer, Alice Roedel, Ralf Dieckmann, Szilvia Vincze, Rita Prager, Antje Flieger, Sascha Al Dahouk, Sylvia Kleta

**Affiliations:** a Department of Biological Safety, German Federal Institute for Risk Assessment, Berlin, Germany; b Department of Biology, Chemistry and Pharmacy, Freie Universität Berlin, Berlin, Germany; c Department of Infectious Diseases, Robert Koch-Institute, Wernigerode, Germany; d Department of Infectious Disease Epidemiology, Robert Koch-Institute, Berlin, Germany; e Department of Internal Medicine III, RWTH Aachen University Hospital, Aachen, Germany

**Keywords:** Listeriosis, outbreak, whole genome sequencing, source tracking, disinfection

## Abstract

Due to its high case fatality rate, foodborne listeriosis is considered a major public health concern worldwide. We describe one of the largest listeriosis outbreaks in Germany with 83 cases of invasive listeriosis between 2013 and 2018. As part of the outbreak investigation, we identified a highly diverse *Listeria monocytogenes* population at a single producer of ready-to-eat meat products. Strikingly, the extensive sampling after identification of a first match between a cluster of clinical isolates and a food isolate allowed for a linkage between this producer and a second, previously unmatched cluster of clinical isolates. Bacterial persistence in the processing plant and indications of cross-contamination events explained long-term contamination of food that led to the protracted outbreak. Based on screening for virulence factors, a pathogenic phenotype could not be ruled out for other strains circulating in the plant, suggesting that the outbreak could have been even larger. As most isolates were sensitive to common biocides used in the plant, hard to clean niches in the production line may have played a major role in the consolidation of the contamination. Our study demonstrates how important it is to search for the origin of infection when cases of illness have occurred (backtracking), but also clearly highlights that it is equally important to check whether a contamination at food or production level has caused disease (forward checking). Only through this two-sided control strategy, foodborne disease outbreaks such as listeriosis can be minimized, which could be a real improvement for public health.

## Introduction

The bacterium *Listeria (L.) monocytogenes* is ubiquitous in nature and the causative agent of human listeriosis, a comparatively rare but potentially life-threatening foodborne disease [1]. The pathogen enters the food chain either through raw products or through contamination of food during processing [2]. Especially ready-to-eat (RTE) products pose a risk for infection [3]. Listeriosis may lead to a self-limiting gastrointestinal disease, to cerebral and bloodstream infections in predominantly immunocompromised patients or to fetal complications in pregnant women [4]. In Germany, the number of notified cases has been constantly increasing from 337 cases in 2011 to 770 cases in 2017, corresponding to an incidence increase from 0.4 to 0.9 cases per 100,000 population [5,6]. In 2018, the number of cases has fallen to 701 again (incidence: 0.8 cases per 100,000 population), with a case fatality rate of 5% [7]. Within the European Union, case fatality was even higher with 15.6% in the same year [8]. Thus, listeriosis represents a considerable burden to society which requires effective surveillance and prevention strategies by close collaboration between public health and food authorities. In Germany, the binational consultant laboratory for *L. monocytogenes* at the German Robert Koch-Institute and the Austrian Agency for Health and Food Safety collects *L. monocytogenes* strains isolated from clinical infections. During the last years, approximately 450 clinical isolates were collected annually, corresponding to approximately two thirds of all listeriosis cases notified in Germany. The National Reference Laboratory (NRL) for *L. monocytogenes*, hosted at the German Federal Institute for Risk Assessment, on the other hand, receives isolates sampled from food and food processing plants.

Molecular surveillance of *L. monocytogenes* using whole genome sequencing (WGS), combined with epidemiological evidence, has greatly facilitated listeriosis outbreak clarification [9–13]. In addition, WGS analysis enables detailed insights into industrial hygiene and forms the basis for in-depth root cause analysis [14].

In our study, we analyzed the diverse *L. monocytogenes* population of a German food processing plant (isolates from food and environment) that was linked to a large long-lasting listeriosis outbreak consisting of two distinct clusters. In addition to backtracking and forward checking, we estimated the virulence potential of the strains circulating in the production facility. Last but not least, we addressed the question of how the contamination has been persisting for years despite periodic hygiene measures.

## Materials and methods

### Bacterial cultivation

*L. monocytogenes* strains were routinely cultured in brain heart infusion (BHI) broth, on BHI agar plates or on sheep blood agar plates at 37°C overnight.

### Pulsed-field gel electrophoresis

Pulsed-field gel electrophoresis (PFGE) was performed according to the PulseNet protocol (https://www.cdc.gov/pulsenet/pdf/listeria-pfge-protocol-92508c.pdf). Restriction patterns were analyzed with BioNumerics, version 7.1 (Applied Maths, Sint-Martens-Latem, Belgium).

### Whole genome sequencing

Genomic DNA was extracted using the GenElute™ Bacterial Genomic DNA Kit (Sigma-Aldrich, St. Louis, MO, United States; clinical isolates) or the QIAamp DNA Mini Kit (Qiagen, Hilden, Germany; non-clinical isolates) following the PulseNet protocol for gram-positive bacteria (https://www.cdc.gov/pulsenet/pdf/pnl32-miseq-nextera-xt.pdf). Extracted DNA was quantified on a fluorescence microplate reader using the Quant-iT™ PicoGreen® dsDNA Assay Kit (Thermo Fisher Scientific, Waltham, MA, United States; clinical isolates) or using the Qubit dsDNA BR Assay Kit with a Qubit 2.0 fluorometer (Invitrogen, Carlsbad, CA, United States; non-clinical isolates). Sequencing libraries from genomic DNA were prepared with the Nextera XT DNA Library Prep Kit (Illumina, San Diego, CA, United States). Sequencing was performed on the MiSeq sequencer in paired-end mode with 2 × 300 bp reads or single-end mode with 1 × 300 bp or 1 × 150 bp reads or on an Illumina HiSeq 1500 sequencer generating 250 bp paired-end reads in a dual flow cell run.

### Sequencing data analysis

#### Data preparation

Sequencing reads were trimmed with Trimmomatic version 0.36 at default parameters [15]. Trimmed reads were either directly used for single nucleotide polymorphism (SNP)-mapping or *de novo* assembled with SPAdes version 3.11.1 [16].

#### Multi locus sequence typing and molecular serogrouping

Multi locus sequence types (MLST STs) as well as corresponding MLST clonal complexes (CCs) and PCR-serogroups were determined from *de novo* assemblies according to the seven house-keeping gene MLST scheme and the PCR-serogrouping scheme, respectively, available at http://bigsdb.pasteur.fr/listeria.

#### Core genome MLST

Core genome MLST (cgMLST) was performed based on assembled genomes in the software Ridom SeqSphere+ (Münster, Germany) with the integrated 1701 genes cgMLST scheme [17]. CgMLST allele coverage of at least 98% was set as quality threshold. CgMLST allelic profiles were imported into BioNumerics version 7.6 to perform single linkage clustering. Isolates with a maximum of ten allele differences from each other were assigned to the same cluster [17]. Trees were visualized and annotated in iTOL version 4 [18].

#### Single nucleotide polymorphism analysis

Trimmed reads were mapped against the sequence of the *L. monocytogenes* strain EGDe (NC_003210.1) using Snippy version 4.0 at default settings [19].

#### In silico screening for antimicrobial resistance and virulence genes

Antimicrobial resistance (AMR) and virulence genes were identified from assembled genomes with ABRicate version 0.8 [20] using the databases ncbi (AMR, 4528 sequences) and vfdb (virulence, 2597 sequences) [21,22], last updated 9 July 2019. To reduce assembly bias, gene coverages were summed up when a gene was split across multiple contigs (visualization with Geneious Prime 2020.0.3). A cutoff of at least 75% gene coverage in total was applied for gene presence.

### Biocide susceptibility testing

Except for benzalkonium chloride (BAC), only active substances of the cleaning agents and disinfectants used in the food processing plant under investigation were tested. BAC was included due to genetically encoded BAC tolerance mechanisms found in the study isolates. Minimum inhibitory concentrations (MICs) of BAC (0.3 to 20 mg/L), sodium hypochlorite (62.5 to 8000 mg/L), peracetic acid (22.3 to 2850 mg/L), hydrogen peroxide (7.8 to 999 mg/L) and phosphoric acid (444.4 to 28440 mg/L) were determined in triplicates as previously described [23]. Biocide susceptibility of nine cgMLST cluster 1 outbreak isolates and two cluster 3 non-outbreak isolates were analyzed exemplarily. Two more isolates were selected for phenotypic testing of BAC tolerance because of their AMR genotype (cluster 9 and 15). The MIC breakpoint ≥4 mg/L was used to classify isolates as BAC tolerant [24,25]. Minimal in-use concentrations of the other biocides were calculated based on manufacturer specifications about biocide concentrations in stock solutions and application concentrations specified in the cleaning and disinfection plan of the processing plant.

### Statistical analysis

Statistical analysis was performed in IBM SPSS Statistics version 21 (IBM, Armonk, NY, United States). Case–control-study was analyzed with Stata 15.0 (StataCorp LLC, TX, United States). In general, analyses with *p*-values lower than 0.05 were considered as statistically significant. In chi-squared tests, *p*-values were adjusted using Bonferroni correction. Strength and direction of a relationship between variables were measured by Spearman correlation.

### Epidemiological analysis

#### Case definition

Outbreak cases were defined as listeriosis patients reported to public health authorities with disease onset in 2013 or later, and isolation of *L. monocytogenes* from normally sterile body fluids revealing either characteristic PFGE profiles (typing method applied before 2015) or belonging to the cgMLST clusters 1 and 2 (typing method applied after 2015, Figure 2). To ensure compatibility of molecular typing results, selected strains with PFGE profiles typical for the outbreak were sequenced retrospectively. In case of affiliation to cgMLST cluster 1 or 2, other isolates showing the same PFGE profile were considered as associated as well.

#### Case–control study

In order to support molecular typing data with epidemiological evidence, a case–control study was conducted in 2017. Recent outbreak cases were asked about their diet in the two weeks prior to disease onset, while earlier cases, from 2016, were asked about general consumption habits during the time of infection (total *n* = 8). Healthy controls from Germany with a similar age and sex distribution as outbreak cases were interviewed by a social research institute using random digit dialling (*n* = 32). Our hypothesis was that the outbreak was caused by the consumption of plastic packaged RTE meatballs. This assumption was compared to consumption of two foodstuffs classically considered at risk for *L. monocytogenes* contamination: plastic-packaged sliced cheese and plastic-packaged smoked fish from the supermarket [1].

## Results

### Epidemiological outbreak description

**Figure 1.**
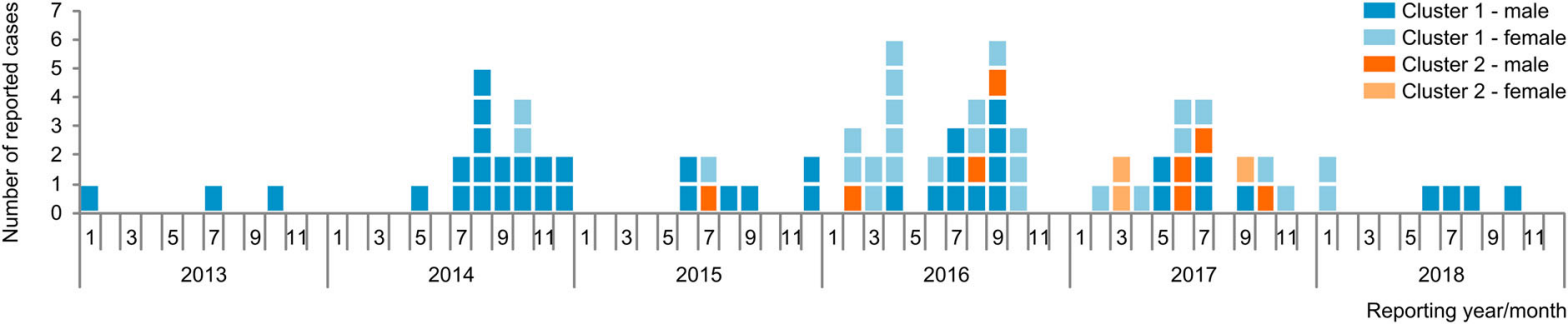
Epidemiological curve of the listeriosis outbreak under investigation. The outbreak comprised 83 cases between 2013 and 2018 and consisted of two distinct core genome MLST clusters (cluster 1, blue, and cluster 2, orange). During the outbreak, a shift from mainly male cases (dark shading) to a balanced ratio between male and female cases (light shading) occurred.

The German surveillance system for *L. monocytogenes* identified an outbreak with 83 invasive listeriosis cases between 2013 and 2018. It consisted of two distinct cgMLST clusters (cluster 1 and cluster 2) which could be traced to the same food processing plant. Because of the common source, both clusters were treated as one outbreak (Figure 1). Cluster 1 had been communicated to European member states on 29/09/2016 via the Epidemic Intelligence Information System (EPIS UI-376), but none of the other participating countries reported cases.

Cluster 1 comprised 72 cases between 2013 and 2018, spread over 12 out of 16 German federal states. Median age of cases in this cluster was 69 years (range 0–96 years) and more male (*n* = 44) than female (*n* = 28) persons were affected. The cluster included six pregnancy-associated cases (mother and child were treated as two separate cases). Five (non-pregnancy-associated) cases died. For three of them, listeriosis was reported as the major cause. Cluster 2 was smaller, included 11 cases between 2015 and 2017 and was reported only from six German federal states. Median age of cases in this cluster was 70 years (range 16–86 years) and again, more male (*n* = 8) than female (*n* = 3) persons were affected. In this cluster, there were no pregnancy-associated cases and no deaths. Cluster 1 reached a peak in 2016 with 26 cases, whereas most cases of cluster 2 appeared in 2017 (*n* = 7) (Figure 1).

From 2013 through 2015, most outbreak cases were male (26 out of 29 cases, 90%). After that the ratio between female and male was almost balanced (26 male versus 28 female cases).

### Clinical *L. monocytogenes* isolates linked to the outbreak

Since clinical *L. monocytogenes* isolates could not always be assigned to a notified case [7], the number of isolates was higher than the number of cases in the outbreak. Some of the isolates were exclusively typed with PFGE, but only sequenced clinical isolates (*n* = 77) were included in our study, with 65 isolates in cgMLST cluster 1, and 12 isolates in cluster 2.

### Source identification and sampling at a food producer

**Figure 2.**
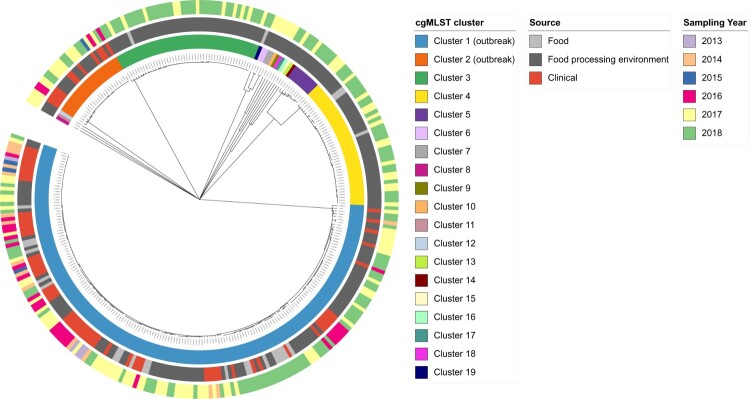
Core genome (cg) MLST-based single linkage clustering of 312 *Listeria monocytogenes* isolates from clinical cases and a single food producer. Colored rings indicate clustering results and metadata of isolates. From inside to outside: cgMLST cluster number, source and sampling year.

During retrospective investigations, an isolate sampled in 2016 from RTE meatballs was found to match the clinical isolates of cgMLST cluster 1 (one allele difference). After checking the NRL database, a second isolate from meatballs of the same producer, sampled in 2014, could be assigned to the same cluster. Both isolates were collected in official controls while the respective product had already been put on the market. Based on these findings, extensive sampling at the food producer was initiated, and isolates from the production facility subsequently matched a second cluster of clinical isolates, namely cluster 2 (Figure 2).

Altogether, 235 non-clinical isolates originating from a single producer were included in our study. Except for the two initial food isolates from 2014 and 2016, isolates were collected in 2017 and 2018. A total of 210 originated from the food processing environment and 25 from food products sampled either at the retail level (*n* = 3) or at the producer level (*n* = 22). Swabs were taken from conveyor belts, pulleys, freezers, accompanying parts like condensate lines or cable ducts, and from gullies. Food isolates originated from RTE meat products such as meatballs or burger patties made from pork, poultry or unknown type of meat and chicken nuggets. The food samples were contaminated below 100 CFU/g [3], except for the one sample from 2014 which contained 3 × 10^4^ CFU/g. Of the 235 non-clinical isolates, 216 (92%) were sampled within self-controls by the producer (21 from food, 195 from food processing environment) and the rest in official controls.

### Case–control study

In order to underpin WGS-based typing results with epidemiological findings, 8 cases and 32 healthy controls were interviewed in 2016 and 2017 concerning their consumption of RTE meatballs, sliced cheese or smoked fish (Table 1). One case could not recall the consumption of plastic packaged RTE meatballs, but exposure to the unpacked product while eating out was not entirely excluded. A second case answered the question on consumption of smoked fish with “I don’t know”. Both cases were classified as not exposed to the respective food category in our analysis. Altogether, six out of eight patients (75%) remembered eating plastic packaged RTE meatballs from retail whereas only two of the 32 controls (6%) did. This corresponds to an odds ratio of 102.4 at a *p*-value of 0.001, indicating a strong association between listeriosis outbreak cases and consumption of plastic-packaged RTE meatballs. In contrast to that, odds ratios between cases and controls for consumption of sliced cheese and smoked fish were 4.4 and 1.3, respectively, and not statistically significant.

**Table 1.** Univariate analysis of factors associated with listeriosis, cgMLST cluster 1, Germany 2017.

Food product (plastic packaged, RTE)	Cases exposed	Controls exposed	Logistic regression, adjusted for age and sex	
	no./total no. (%)	no./total no. (%)	Odds ratio (95% CI^a^)	*p*-value
Meatballs	6/8 (75)^b^	2/32 (6)	102.4 (7.0–1509.6)	0.001
Sliced cheese	6/8 (75)	17/32 (53)	4.4 (0.5–42.0)	0.200
Smoked fish	1/8 (13)^b^	3/32 (9)	1.3 (0.1–14.7)	0.851

^a^CI = confidence interval; ^b^A case in this category has been classified as not exposed due to unclear information.

### Molecular typing and cluster analysis of strains

The 312 isolates included in our study were assigned to 11 different MLST CCs spanning four serogroups (IIb, IIa, IVb and IIc, with decreasing prevalence). The majority (>95%) of isolates fell into CC5 (*n* = 176), CC121 (*n* = 50), CC31 (*n* = 46) and CC7 (*n* = 24). Clinical isolates were either CC5 (*n* = 65, serogroup IIb) or CC7 (*n* = 12, serogroup IIa).

The isolates fell into 7 cgMLST clusters and 12 singletons (containing only one non-clinical isolate) (Figure 2). The outbreak-associated cgMLST clusters 1 and 2 contained 176 and 24 isolates, respectively. Cluster 1 was composed of 65 clinical isolates, 20 isolates from food and 91 isolates from food processing environment. All isolates in this cluster were closely related with an overall allelic distance between 0 and 18 (median 7). The close genetic relatedness of isolates in this cluster could be confirmed by SNP analysis (overall SNP distance 0–60, median 10). Cluster 2 contained only 24 isolates, 12 of clinical origin and 12 from food processing environment. Allelic differences ranged between 0 and 8 (median 2). Corresponding SNP distances were between 0 and 8 with a median of 3. With an allelic distance of 1633, the genetic difference between cluster 1 and cluster 2 was large. The two outbreak clusters did not show sub-clustering according to the source of isolates or the time of sampling (Figure 2). For the other cgMLST clusters of the non-clinical isolates, no match to clinical isolates from Germany could be found.

Four cgMLST clusters (cluster 1, 3, 4 and 19) contained isolates from RTE food products and three of those (cluster 1, 3 and 4) also isolates from food processing environment. In the remaining 15 cgMLST clusters, non-clinical isolates originated only from the food processing environment. Four cgMLST clusters (cluster 2–5) contained isolates which have been sampled over a period of 9–12 months, in 2017 and 2018. Cluster 1 has been detected over four years.

### Virulence genes

**Figure 3.**
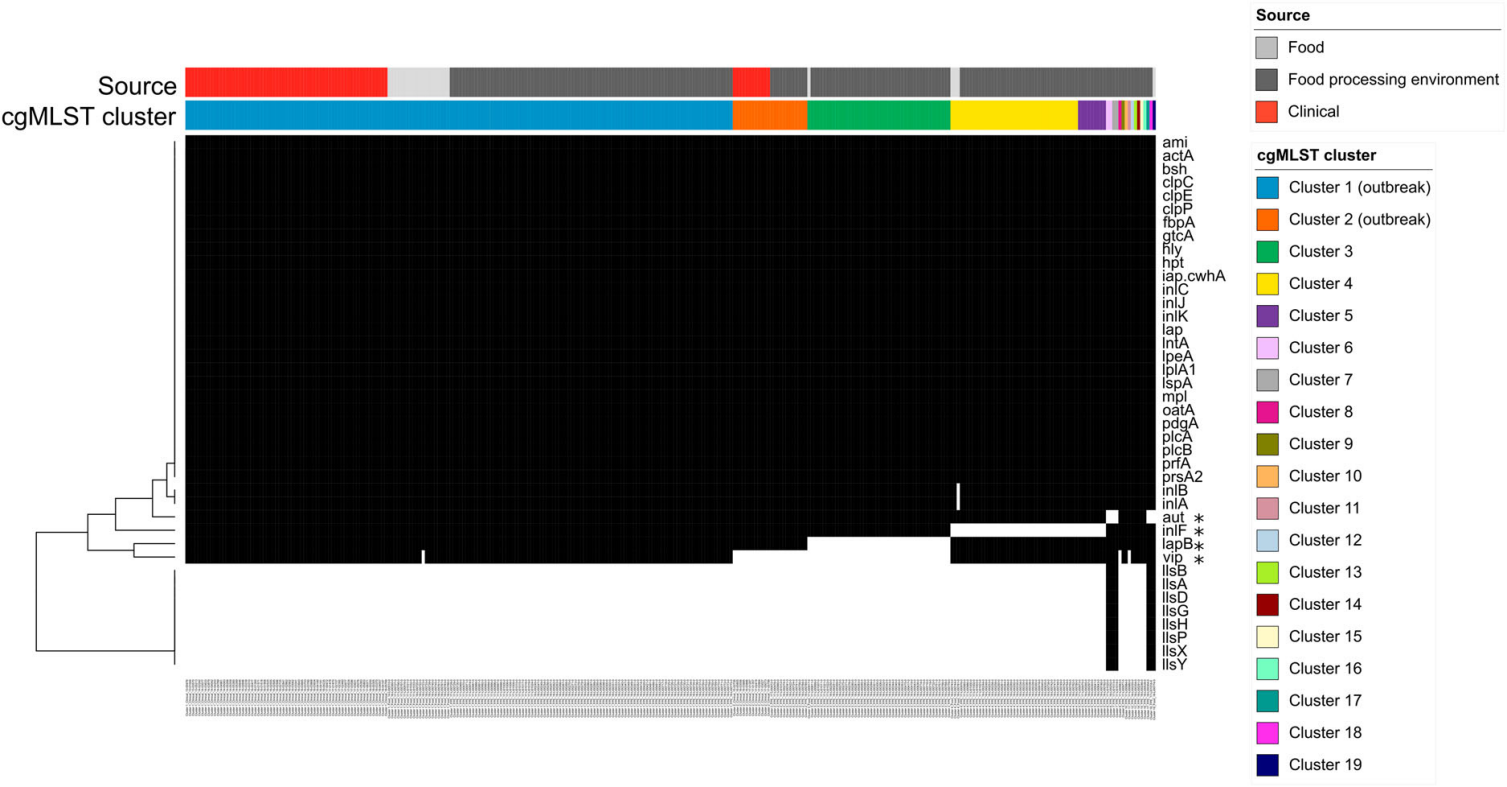
Heatmap of *in silico* detected virulence genes. Black: gene present; white: gene absent. Isolates are sorted by source and by cgMLST cluster number. A set of 26 virulence genes was present in all 312 study isolates. Genes positively associated with the outbreak clusters are marked by an asterisk. CgMLST clusters 9, 10, and 12–16 had the same virulence factor composition as outbreak cluster 1. Isolates in the clusters 8 and 11 were lacking the *vip* gene and were hence identical to outbreak cluster 2.

A total of 40 different virulence genes could be identified in the 312 isolates (Figure 3). A single isolate contained 29–39 virulence genes (median 32). Virulence gene counts were significantly different between outbreak and non-outbreak clusters (Mann–Whitney-U test, *p *< 0.001). A set of 26 virulence genes was found in all isolates.

Genes positively associated with the two outbreak clusters were *aut, inlF, lapB* and *vip* (correlation 0.2, 0.58, 0.56, 0.34, respectively; 1-tailed *p *< 0.01). LIPI-3 (*llsAGHXBYDP*, [26]) was only detected in non-outbreak clusters (cluster 6, 7 and 17–19) with a correlation of 0.2 (1-tailed *p *< 0.01).

Clusters 9, 10, and 12–16 had the same virulence factor composition as outbreak cluster 1. The virulence factors of cluster 8 and cluster 11 isolates were identical to outbreak cluster 2 isolates lacking the *vip* gene.

### Antimicrobial susceptibility

**Figure 4.**
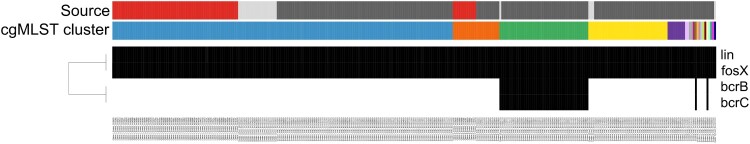
Heatmap of *in silico* detected antimicrobial resistance genes. Black: gene present; white: gene absent. Isolates are sorted by source and by cgMLST cluster number (for legend see Figure 3). All 312 study isolates contained the *lin* gene (lincomycin resistance) and the *fosX* gene (fosfomycin resistance). Only 48 isolates of three different cgMLST clusters (cluster 3, 9 and 15) additionally contained the benzalkonium chloride tolerance genes *bcrB* and *bcrC*.

In all 312 isolates, the AMR genes *lin*, coding for the lincomycin resistance ABC-F type ribosomal protection protein, and *fosX*, coding for the fosfomycin resistance thiol transferase FosX, were identified (Figure 4).

In 48 isolates of three different cgMLST clusters (3, 9 and 15), none of them associated with clinical cases, the BAC tolerance genes *bcrB* and *bcrC* were found [27] (Figure 4). At least one representative isolate per *bcrB-bcrC* containing cgMLST cluster was phenotypically tested and confirmed as BAC tolerant (MIC values ranging from 5 to 10 mg/L). Representative isolates of the outbreak cluster 1, lacking *bcrB* and *bcrC*, showed a BAC MIC value of 2.5 mg/L and were classified as susceptible. MIC values of the other biocides tested did not differ between isolates. MIC of sodium hypochlorite was 500 mg/L, of peracetic acid 356.3 mg/L, of hydrogen peroxide 249.8 mg/L and of phosphoric acid 3555 mg/L. All MIC values were below in-use concentrations.

## Discussion

With 83 confirmed cases, the outbreak described in our study represents one of the largest listeriosis outbreaks identified in Germany since cgMLST has been implemented for molecular surveillance [28]. From 2017 to 2018, for the first time after the introduction of mandatory reporting of human listeriosis, the number of notified cases in Germany declined [5,7]. Using the potential of WGS to resolve and to stop large outbreaks [13,29] might have already made a decisive contribution towards reducing the burden of listeriosis in Germany. More males than females were affected within the outbreak described here. This is in line with the general trend of a significantly higher incidence rate of listeriosis among men in Germany [5,7,30–32]. Interestingly, there was a gender shift of cases during the outbreak. General changes in consumption habits as well as the introduction of novel food products could have played a role. Both factors are likely to influence the ingested dose and hence the relevant dose–response relationship for a consumer group of interest – in this case women [33]. The age distribution was typical for a listeriosis outbreak, representing the population groups at risk: elderly people (older than 69 years of age), pregnant women (women of fertile age), and newborns.

In the first outbreak cluster, the entire hypothetical transmission chain could be traced back from clinical cases to food product to food processing environment due to the high genetic relatedness of isolates. Indeed, the cgMLST-based suspicion that RTE meatballs were the causative food vehicle for listeriosis infections could be epidemiologically confirmed by a case–control study. In the second outbreak cluster, the food product was missing in the transmission chain. However, as soon as the contamination in the food processing environment was detected and eliminated, further contamination of food products and hence transmission to consumers was effectively prevented. Consequently, forward checking, along with backtracking, and the resulting recall of products from the market as well as stopping of the production, finally terminated the outbreak.

Because of the great variety of *L. monocytogenes* strains at the producer, the question arose as to why only two of them have caused human infections although probably more of them had reached consumers. One possible explanation could be that strains differ in pathogenicity. In line with a recent French study showing associations of certain MLST CCs with either infection or food [34], CC121 was the second most common MLST CC in our non-clinical isolates. The two outbreak clusters, however, belonged to CC5 and CC7, which were classified as intermediate and even rarely responsible for clinical cases, respectively. Analysis of clinical *L. monocytogenes* isolates from Germany [28] had confirmed that CC5 and CC7 have not frequently been associated with listeriosis cases so far. To gain a deeper insight into strain pathogenicity, we screened the genomes of all isolates for known virulence factors [21]. The genes *aut*, *vip*, *inlF* and *lapB*, which are all critical for host cell entry [35–37], were found to be positively associated with outbreak isolates and thus appeared to be involved in a strain’s ability to infect humans. However, those virulence genes were also found in non-outbreak clusters including infection-associated MLST CCs, such as CC1 and CC6 [34]. Additionally, LIPI-3 (*llsAGHXBYDP*, [26]) was detected in non-outbreak clusters, a genetic island which is linked to increased invasiveness of *L. monocytogenes* [38]. Last but not least, pathogenicity is a multifactorial process, and cannot merely be derived from the presence or absence of virulence genes [39]. In summary, the variety of potentially pathogenic strains circulating at the producer, as already described in other food processing plants [40], clearly shows that selectively removing the source of contamination for one specific outbreak cluster is neither sufficient nor sustainable. Instead, the entire *L. monocytogenes* population in a food processing plant must be controlled and eliminated to not only stop present outbreaks but to also prevent future ones.

The high diversity of the *L. monocytogenes* population found in the food processing plant is not necessarily due to an extraordinary extent of contamination, but very likely results from extensive sampling (“Who seeks shall find”). Nevertheless, it highlights the problem of recurring *L. monocytogenes* contamination at the producer and insufficiently established hygiene measures. Strains of at least five of the *L. monocytogenes* clusters were persistent in the food processing plant and have been detected for nine months to four years. This long period of time may explain the comparatively large allele and SNP distance [12,41–43] between the epidemiologically linked isolates in our study. Furthermore, in three of those clusters, strains were found in both, food and food processing environment, verifying cross-contamination events. Detection of twelve cgMLST singletons showed a snapshot which does not necessarily exclude persistence of these strains. This observation may also provide evidence for periodic entry events of *L. monocytogenes* into the production line, for example via raw meat from various suppliers.

To gain a better understanding of the reason for long-lasting persistence of *L. monocytogenes* strains in the food processing plant, we screened for AMR genes. Inadequate disinfection practices may expose bacteria to sub-lethal biocide concentrations and thus select for tolerant strains which may then persist in niches [44]. As *L. monocytogenes* is known to be naturally resistant to lincomycin and fosfomycin [45–47], full length detection of these two AMR genes in all isolates illustrated the effectiveness of our in silico screening method. Forty-eight out of 235 *L. monocytogenes* isolates (20%) from food or food processing environment, found in cgMLST clusters 3, 9 and 15, carried the BAC tolerance genes *bcrB* and *bcrC*. These figures are in agreement with a recent study that found a prevalence rate of BAC tolerance of 16% in 93 isolates from German food processing environments collected from 2008 through 2016 [23]. However, since November 2016, BAC has been listed as an unapproved disinfectant and preservative in the EU (implementation decision 2016/1950), and most of our study population and importantly, all isolates in outbreak clusters, were susceptible. All tested isolates exhibited MIC values lower than the in-use concentrations of biocides in the cleaning agents and disinfectants applied in the high care area of the food processing plant. We therefore assume that all substances were suitable for cleaning and disinfection if hygiene measures met the guidelines. Hence, retention in hard-to-reach and consequently hard-to-clean niches may have played a major role in the establishment of persistence. Indeed, *L. monocytogenes* contamination was found in such niches along production lines and included surfaces in high risk areas where previously heat-treated meat products were chilled before packaging. The knowledge gained about hotspots of contamination should help to improve cleaning regimes including periodic disassembly of production lines and/or to redesign the manufacturing equipment so that hard-to-clean areas are minimized.

As observed in other studies [48,49], neither adjustment of the hygiene management concepts, nor infrastructural changes were successful to get the *L. monocytogenes* contamination at the producer under control. As a result, the entire processing plant was shut down in autumn 2018. With the last clinical case in October 2018, the outbreak was considered as terminated.

In the beginning of 2019, however, an isolate from a second producer located in another German federal state matched outbreak cluster 1 of our study according to cgMLST. Throughout the year, 16 isolates from this second producer, 3 from RTE meat products and 13 from the food processing environment, were found to be highly genetically related to the isolates of the outbreak. Both producers did not have a direct supply relationship and, in so far as this is known, neither equipment nor staff has been transferred between them. However, they shared some of their suppliers, supporting the hypothesis that the outbreak strain has been introduced via contaminated raw animal products in both plants. On the one hand, this shows that the search for an outbreak source does not necessarily end at the level of final food processing but needs to be extended to the level of slaughterhouses and cutting plants to really address the root of the problem. Ultimately, this means that not only sharing of sequencing data is needed, but that integration of information on commodity chains into a common database would be important as well. On the other hand, it also shows the need for epidemiological investigations in addition to molecular surveillance. Despite the high genetic relatedness of the new isolates to the former outbreak, no further clinical cases related to the outbreak have been reported up to the date of publication.

### Conclusion

In order to prevent listeriosis cases before they occur, we should not only carry out outbreak detection, but also set a focus on expansion of the data set available for WGS-matching. One important approach for that purpose would be to intensify regular monitoring in the companies. Preferably, this is largely implemented in the form of company’s own checks, so that contaminated food products are detected early enough and do not enter the market. Overall, backtracking and forward checking along the entire food chain must go hand in hand to protect the public from zoonotic pathogens. These terms are inspired by the area of artificial intelligence, where forward checking is used as a look ahead strategy during backtracking [50]. A common database of molecular typing results may solve this problem automatically, since it enables real-time comparison in both directions. Only through this two-sided control strategy, foodborne disease cases can be prevented.

## Supplementary Material

EMI_Supplement_ENA-Accessions.xlsx

## Data Availability

The sequence data for this study have been deposited in the European Nucleotide Archive (ENA) at EMBL-EBI under accessions listed in Supplementary Table 1.
